# Effect of Opaganib on Supplemental Oxygen and Mortality in Patients with Severe SARS-CoV-2 Based upon FIO_2_ Requirements

**DOI:** 10.3390/microorganisms12091767

**Published:** 2024-08-26

**Authors:** Fernando Carvalho Neuenschwander, Ofra Barnett-Griness, Stefania Piconi, Yasmin Maor, Eduardo Sprinz, Nimer Assy, Oleg Khmelnitskiy, Nikita V. Lomakin, Boris Mikhailovich Goloshchekin, Ewelina Nahorecka, Adilson Joaquim Westheimer Calvacante, Anastasia Ivanova, Sergey Vladimirovich Zhuravel, Galina Yurevna Trufanova, Stefano Bonora, Amer Saffoury, Ami Mayo, Yury G. Shvarts, Giuliano Rizzardini, Rogerio Sobroza de Mello, Janaina Pilau, Alexey Klinov, Benjamin Valente-Acosta, Oleg Olegovich Burlaka, Natalia Bakhtina, Maskit Bar-Meir, Ivan Nikolaevich Shishimorov, Jose Oñate-Gutierrez, Cristian Iván García Rincón, Tatiana Ivanovna Martynenko, Ludhmila Abrahão Hajjar, Ana Carolina Nazare de Mendonca Procopio, Krzysztof Simon, Walter Gabriel Chaves Santiago, Adam Fronczak, Conrado Roberto Hoffmann Filho, Osama Hussein, Vladimir Aleksandrovich Martynov, Guido Chichino, Piotr Blewaska, Jacek Wroblewski, Sergio Saul Irizar Santana, Andres Felipe Ocampo Agudelo, Adam Barczyk, Rachael lask Gerlach, Eppie Campbell, Aida Bibliowicz, Reza Fathi, Patricia Anderson, Gilead Raday, Michal Klein, Clara Fehrmann, Gina Eagle, Vered Katz Ben-Yair, Mark L. Levitt

**Affiliations:** 1 Núcleo de Pesquisa Clínica—Hospital Vera Cruz, Belo Horizonte 30190-130, Brazil; fcn2709@gmail.com; 2 Bioforum Ltd., Ness Ziona 7403636, Israel; ofra.barnett@bioforumgroup.com (O.B.-G.);; 3 Ospedale A. Manzoni, 23900 Lecco, Italy; s.piconi@asst-lecco.it; 4 Infectious Disease Unit, E. Wolfson Medical Center, Holon 58100, Israel; yasminm@wmc.gov.il; 5 Faculty of Medical and Health Sciences, Tel-Aviv University, Tel Aviv 6997801, Israel; 6 Hospital de Clínicas de Porto Alegre, Porto Alegre 90035-903, Brazil; esprinz@hcpa.edu.br; 7 Western Galilee Medical Center, Nahariya 221001, Israel; nimera@gmc.gov.il; 8 Saint-Petersburg State Budget Healthcare Institution “City Pokrovskaya Hospital”, Saint-Petersburg 199106, Russia; oleg.khmelnitskiy@gmail.com; 9 Russian Medical Academy of Continuous Professional Education of the Ministry of Health of the Russian Federation, Moscow 121359, Russia; lomakinnikita@gmail.com; 10 Saint-Petersburg SBHI City Hospital 15, Saint-Petersburg 197110, Russia; bgoloschekin@yandex.ru; 11 Zespół Opieki Zdrowotnej, 59-700 Bolesławiec, Poland; ewelina-straszak@wp.pl; 12 CEMEC—Centro Multidisciplinar de Estudos Clinicos LTDA, São Bernardo do Campo 09715-090, Brazil; drwesth@gmail.com; 13 State Budgetary Institution of Ryazan Region “Regional Clinical Hospital”, Ryazan 390026, Russia; nastya_doctor@list.ru; 14 N.V. Sklifosovsky Research Institute for Emergency Medicine of Health Department of Moscow, Moscow 129090, Russia; zhsergey5@gmail.com; 15 State Budgetary Healthcare Institution of the Tver Region “Regional Clinical Hospital”, Tver 170100, Russia; trufanovagala@mail.ru; 16 Ospedale Amedeo di Savoia, 10149 Torino, Italy; stefano.bonora@unito.it; 17 Nazareth Hospital EMMS, Nazareth 19152, Israel; amer_saffoury@nazhosp.com; 18 Assuta Medical Center Ashdod, Ashdod 7747629, Israel; amim@assuta.co.il; 19 Clinical Hospital n.a-S.R. Mirotvortseva SSMU, Saratov 410012, Russia; shwartz58@yandex.ru; 20 Ospedale Luigi Sacco, 20157 Milan, Italy; giuliano.rizzardini@asst-fbf-sacco.it; 21 Hospital Nossa Senhora da Conceição de Tubarão, Centro de Pesquisas Clínicas do Hospital Nossa Senhora da Conceição, Tubarão 88701-160, Brazil; rogerio.sobroza@gmail.com; 22 Hospital de Clínicas de Passo Fundo, Cento de Pesquisa Clínica, Passo Fundo 99010-260, Brazil; jpilau@hotmail.com; 23 Kirovsk Interregional Hospital, Leningrad 187342, Russia; klinov_md@inbox.ru; 24 Hospital ABC, Mexico City 05348, Mexico; benjamin.valente-acosta1@alumni.lshtm.ac.uk; 25 Saint-Petersburg State Budgetary Healthcare Institution “City Aleksandrovskaya Hospital”, Saint-Petersburg 193312, Russia; burlaka@list.ru; 26 State Regional Budgetary Healthcare Institution Murmansk Regional Clinical Hospital, Murmansk 183032, Russia; nv_bahtina@mail.ru; 27 Shaare Zedek Medical Center, Jerusalem 9103102, Israel; bmaskit@szmc.org.il; 28 Department of Pediatrics and Neonatology, Institute of Medical and Physiotherapy, Federal State Budgetary Educational Institution of Higher Education Volgograd State Medical University, Volgograd 400087, Russia; drshishimorov@gmail.com; 29 Centro Medico Imbanaco de Cali, Valle del Cauca 760042, Colombia; jose.onate@imbanaco.com.co; 30 Unidad de Transferencia y Ensayos Clínicos Clinica Universitaria Bolivariana, Medellin 050021, Colombia; cristianivan.garcia@upb.edu.co; 31 Regional State Budgetary Healthcare Institution “City Hospital No. 5, Barnaul”, Barnaul 656045, Russia; ti_martynenko@mail.ru; 32 Instituto do Coração do Hospital das Clínicas, Faculdade de Medicina, Universidade de São Paulo, Andar 05403-900, Brazil; ludhmilah@gmail.com; 33 Hospital Felicio Rosso, Belo Horizonte 30110-934, Brazil; anacprocopio@hotmail.com; 34 Szpital Specjalistyczny im. Gromkowskiego, 51-149 Wrocław, Poland; krzysimon@gmail.com; 35 Sociedad de Cirugía de Bogotá Hospital de San José, Bogota 110821, Colombia; wgchs1973@gmail.com; 36 Centrum Onkologii w Lodzi, Oddzial COVID-19, 93-510 Lodz, Poland; adamum@op.pl; 37 Hospital Regional Hans Dieter Schmidt, Joinville 89227-680, Brazil; conrado@corsanus.com.br; 38 Ziv Medical Center, Safed 13100, Israel; osama.h@ziv.health.gov.il; 39 State Budgetary Healthcare Institution of the Tver Region “Regional Clinical Hospital”, Ryazan 390026, Russia; dr.martinov@mail.ru; 40 Azienda Ospedaliera SS, 15121 Alessandria, Italy; guido.chichino@ospedale.al.it; 41 Szpital Rejonowy w Raciborzu, 47-400 Racibórz, Poland; 42 Szpital Wojewódzki im. Mikołaja Kopernika, 75-581 Koszalin, Poland; jw@swk.med.pl; 43 Hospital Civil de Culiacan, Sinaloa 80030, Mexico; nefro.irizar@gmail.com; 44 Fundacion Hospitalaria San Vicente, Antioquia 050001, Colombia; andres.ocampo@sanvicentefundacion.com; 45 Department of Pneumology, School of Medicine in Katowice, Medical University of Silesia, 40-055 Katowice, Poland; adagne@icloud.com; 46 RedHill Biopharma Ltd., Tel-Aviv 6473921, Israel; rachael.lask@gmail.com (R.l.G.); eppie@redhillbio.com (E.C.); abf0306@gmail.com (A.B.); reza@redhillbio.com (R.F.); patricia@redhillbio.com (P.A.); gilead@redhillbio.com (G.R.); vered.kby@gmail.com (V.K.B.-Y.); 47 CEEF Solutions Beaconsfield, Pointe-Claire, QC H9S 4L7, Canada; clara@redhillbio.com; 48 G.E.T. Pharma Consulting, LLC, Lumberville, PA 18933, USA; ginaeagle@getpharmaconsulting.com

**Keywords:** opaganib, COVID-19, sphingosine kinase 2, ABC294640, SARS-CoV-2 pneumonia, trial registration number: NCT04467840

## Abstract

Once a patient has been diagnosed with severe COVID-19 pneumonia, treatment options have limited effectiveness. Opaganib is an oral treatment under investigation being evaluated for treatment of hospitalized patients with severe COVID-19 pneumonia. A randomized, placebo-controlled, double-blind phase 2/3 trial was conducted in 57 sites worldwide from August 2020 to July 2021. Patients received either opaganib (n = 230; 500 mg twice daily) or matching placebo (n = 233) for 14 days. The primary outcome was the proportion of patients no longer requiring supplemental oxygen by day 14. Secondary outcomes included changes in the World Health Organization Ordinal Scale for Clinical Improvement, viral clearance, intubation, and mortality at 28 and 42 days. Pre-specified primary and secondary outcome analyses did not demonstrate statistically significant benefit (except nominally for time to viral clearance). Post-hoc analysis revealed the fraction of inspired oxygen (FIO_2_) at baseline was prognostic for opaganib treatment responsiveness and corresponded to disease severity markers. Patients with FIO_2_ levels at or below the median value (≤60%) had better outcomes after opaganib treatment (n = 117) compared to placebo (n = 134). The proportion of patients with ≤60% FIO_2_ at baseline that no longer required supplemental oxygen (≥24 h) by day 14 of opaganib treatment increased (76.9% vs. 63.4%; nominal *p*-value = 0.033). There was a 62.6% reduction in intubation/mechanical ventilation (6.84% vs. 17.91%; nominal *p*-value = 0.012) and a clinically meaningful 62% reduction in mortality (5.98% vs. 16.7%; nominal *p*-value = 0.019) by day 42. No new safety concerns were observed. While the primary analyses were not statistically significant, post-hoc analysis suggests opaganib benefit for patients with severe COVID-19 requiring supplemental oxygen with an FIO_2_ of ≤60%. Further studies are warranted to prospectively confirm opaganib benefit in this subpopulation.

## 1. Introduction

Severe Acute Respiratory Syndrome Coronavirus 2 (SARS-CoV-2) has caused almost 7 million deaths by coronavirus disease-19 (COVID-19) worldwide [1]. Patients infected with SARS-CoV-2 may progress to acute respiratory distress syndrome (ARDS) followed by death. The global need for therapeutics to safely treat severe COVID-19 has become more urgent with recent increases in breakthrough SARS-CoV-2 infections due to emerging viral variants and continued vaccine hesitancy [2]. 

Currently, intravenous remdesivir, tocilizumab, baricitinib, and anakinra (in certain patients) are the only therapeutics approved for use in cases of severe COVID-19 by the Food and Drug Administration (FDA) [3]. However, other more recent studies have not found remdesivir to be effective [4]. 

Opaganib ([3-(4-chlorophenyl)-adamantane-1-carboxylic acid (pyridin-4-ylmethyl)amide, hydrochloride salt]) is an orally available, first-in-class, substrate-competitive sphingosine kinase 2 (SK2) selective inhibitor [5]. SK2 is a molecular target due to its critical role in sphingolipid metabolism, including as a host factor in the replication-transcription complex (RTC) of certain RNA viruses [6]. 

Preclinical studies of the opaganib in Chikungunya virus, a (+)ssRNA virus (like SARS-CoV-2), determined that inhibition of SK2 with opaganib markedly inhibited viral transcription [6]. In addition, our pre-clinical studies have demonstrated that opaganib is a potent inhibitor of SARS-CoV-2 replication (data on file). Opaganib also inhibits both dihydroceramide desaturase and glucosylceramide synthase, both of which may play a role in its anti-SARS-CoV-2 activity [7,8,9]. Importantly, SK2 is likely unaffected by viral mutations, including those of the viral spike protein. 

In a *Pseudomonas aeruginosa* pneumonia model, opaganib decreased lung injury and associated inflammation [10]. Thus, inhibition of SK2 by opaganib may provide therapeutic benefit in reducing lung inflammatory injury.

Given the potential anti-viral and anti-inflammatory properties of opaganib, we conducted a small phase 2a clinical trial to evaluate opaganib for the treatment of SARS-CoV-2 infection in hospitalized patients with COVID-19 pneumonia that suggested clinical benefit in this population [11]. We subsequently evaluated a 14-day course of opaganib therapy for reduction in the need for supplemental oxygen and improvement in the clinical status of hospitalized patients with severe COVID-19 pneumonia (ClinicalTrials.gov Identifier: NCT04467840). 

## 2. Methodology

### 2.1. Study Settings and Trial Design

This clinical trial was a multicenter, phase 2/3, randomized, double-blind, placebo-controlled opaganib treatment study involving patients diagnosed with COVID-19 infection defined by eligibility criteria targeting World Health Organization (WHO) Ordinal Scale for Clinical Improvement level 5. Between August 2020 and July 2021, 475 patients were recruited (of which 463 received at least one dose of the investigational product—303 male and 160 female) at 57 locations in 7 countries (NCT04467840) [12]. All patients were 18 to 80 years of age (inclusive), tested positive for SARS-CoV-2 infection by RT-PCR, had pneumonia secondary to SARS-CoV-2 based on radiographic evidence via chest X-ray or CT scan, and required supplemental oxygen as high flow, positive pressure ventilation, or a non-rebreather face mask at high oxygen concentrations. There were no limits on the number of days from symptom onset. For a short period, patients with simple face masks and an oxygen flow rate greater than 5 L/min were allowed, due to a lack of adequate high flow devices at several sites. Patients with serious co-morbidities or receiving drugs likely to interact with opaganib were excluded. Patients were required to be hospitalized at baseline day 1. Patient-reported race and ethnicity categories were collected as part of the demographic characteristics. Investigators reviewed symptoms, risk factors, and other inclusion and exclusion criteria prior to enrollment. The trial complied with the Declaration of Helsinki, the International Conference on Harmonization Guidelines for Good Clinical Practice, and applicable local regulations. The protocol was approved by the ethics committees at all participating centers. All patients provided written informed consent before study entry. 

### 2.2. Randomization and Intervention

Patients received local standard of care (SoC) and were randomized to receive either 2 × 250 mg opaganib capsules (500 mg) every 12 h or a matching placebo. Randomization and supply of the study drugs were managed through Interactive Response Technology (IRT). This study used block randomization in which patients were randomized into the two treatment arms, opaganib and placebo, in a 1:1 ratio at the study level and not by the country or site level. 

Stratification was performed based on:

1. Whether the patients met three or more high-risk parameters for COVID-19 outcomes at baseline. This was determined by the following eight parameters: age at screening ≥ 60 years; male; HbA1c at screening ≥ 6.5 or on active treatment with insulin or oral hypoglycemics; hypoxemia without commensurate increased work of breathing; known underlying chronic lung disease; known cardiovascular disease or hypertension; BMI ≥ 28.0 kg/m^2^; known renal disease.
2. Whether SoC treatment has established efficacy (yes or no). A standard of care (SoC) treatment with established efficacy was defined by Emergency Use Authorization or full approval granted by either the US Food and Drug Administration (FDA), the European Medicines Agency (EMA), or the Medicines and Healthcare Products Regulatory Agency (MHRA). The proven effective therapies for the purpose of this study were adjusted as new data emerged and were documented and shared with study personnel regularly. The following treatments were included in the list of proven effective therapies at the time the study was being conducted: dexamethasone, remdesivir, COVID-19 convalescent plasma, and baricitinib in combination with remdesivir.

Treatment assignments were blinded to the patient, investigator, and hospital staff, as well as the sponsor. The study drug was administered for 14 days; participants were followed for 42 days from their first dose. 

### 2.3. Primary, Secondary, and Exploratory Outcomes

The primary outcome was a measurement of the proportion of patients no longer requiring supplemental oxygen by the end of treatment day 14, which was defined per patient in a binary manner as “success” or “failure.” Recurrent need for oxygen after day 14 and death or withdrawal from this study were considered failures regardless of previous success. The primary objective of this clinical trial was to evaluate the proportion of patients no longer requiring supplemental oxygen by day 14 for at least 24 h, with a 15% raw difference being targeted. A total of nine efficacy secondary outcomes were evaluated. Four outcome measures involved consideration of the need for oxygen supplementation, including (1) two levels or greater improvement in the WHO Ordinal Scale for Clinical Improvement by day 14, (2) time until recovery as defined by improvement to a score of 3 or less on the WHO scale, (3) time until transition to low oxygen flow via nasal cannula from high oxygen flow via nasal cannula or positive pressure ventilation at baseline, and (4) the proportion of patients requiring intubation and mechanical ventilation by day 42. In addition, both time to discharge from the hospital and mortality at days 28 and 42 following the initiation of treatment were measured. An additional outcome focused on general health, including measures of the proportion of patients transitioning from a fever at baseline (>38.0 °C, 100.4 °F) to being afebrile (<37.2 °C, 99 °F) by day 14. Two additional endpoints focused on infection were (1) measures of the proportion of patients with two consecutive negative swabs for SARS-CoV-2 by RT-PCR at day 14 and (2) the time until two consecutive negative swabs through day 14. Exploratory outcomes included the mean change in systemic markers of inflammation (D-dimer, cardiac troponin, C-reactive protein, lactate dehydrogenase, and ferritin) as well as lymphocyte count from baseline at day 14 were to be evaluated. Time until recovery, as defined by improvement to a score of 1 or less on the WHO Ordinal Scale for Clinical Improvement, the percentage of patients no longer requiring supplemental oxygen for at least 24 h by day 7, and the time until a 50% reduction of supplemental oxygen requirement for the subset of patients who did not receive positive pressure ventilation (non-invasive or invasive) were all evaluated in the opaganib versus placebo arm. Adverse events (AEs) and serious AEs (SAEs) were evaluated with respect to vital signs, laboratory parameters (chemistry and hematology), electrocardiograms, and incidence rates of treatment-emergent AEs (TEAEs) and SAEs. 

### 2.4. Sample Size Calculation

The sample size calculation was based on powering the study with respect to the primary analysis of the primary efficacy endpoint of proportions of patients no longer requiring supplemental oxygen for at least 24 h by day 14. It was assumed that the treatment success rate at 14 days in the control arm would be 40% and that opaganib is expected to provide an absolute 15% increase of this rate, to a success rate of 55%. A total of 464 subjects provides 90% power to detect the assumed difference in success rate, using the chi square test, at a two-sided α = 0.05 level of significance. This sample size calculation took into account a planned non-binding futility analysis to be performed after at least 135 patients in the study have been evaluated for the primary endpoint.

### 2.5. Statistical Analysis

Eligible patients (n = 464) were to be randomized to double-blind treatment. An opaganib intent to treat (ITT) (n = 232) and placebo standard of care (n = 232) were met. This sample size calculation was based on powering this study to the primary analysis of patients no longer requiring supplemental oxygen for at least 24 h by day 14. This endpoint was chosen as improvement in hospitalized patients not requiring invasive mechanical intubation is based on oxygen requirements specified in the WHO Ordinal Scale for Clinical Improvement. This calculation assumed an opaganib treatment success corresponding to an elimination of supplemental oxygen in at least 15% more of the patients than in the placebo arm. The 464 patients provided 90% power to detect the assumed difference in success rate using a chi square test with a two-sided α = 0.05 level of significance. Efficacy endpoints were analyzed for the modified ITT (mITT) population (patients who received at least one dose of the study drug). Safety evaluations were performed for the safety population (patients who took at least one dose of study drug). A post-hoc analysis was performed for evaluating COVID-19 severe pneumonia patients entering this study with ≤60% FIO_2_. The primary endpoint analysis included a Cochran–Mantel–Haenzel test to compare the proportion of success between the two groups, using the study stratification factors as used for randomization and the corresponding stratified risk difference estimate along with a 95% confidence interval. Time to event secondary endpoints were tested using the Log Rank test stratified by study stratification factors used for randomization, with treatment effect summarized in terms of Hazard Ratio (HR, Opaganib to placebo) and its 95% confidence interval (estimated by a Cox proportional hazards regression model with treatment group as an explanatory variable and stratification factors as covariates) and described using a Kaplan–Meier estimator. Binary secondary endpoints were analyzed similarly to methods used for the primary analysis. All analyses were performed using SAS^®^ software, version 9.4.

The Data Safety Management Board endorsed permanent closure of site 114 after review of unblinded data that showed very high within-site mortality rate due to the same outcome of “worsening pneumonia” for 8/15 (53.3%) patients. After database lock, it was noted that of the 15 patients enrolled at site 114, 12 patients were randomized to the opaganib arm and 3 patients to the placebo arm, all of whom were in the >60% baseline FIO_2_ subpopulation, with 7/12 patients in the opaganib arm and 1/3 patients in the placebo having an SAE of worsening pneumonia with an outcome of death. This anomaly possibly introduced a confound for both the mITT and FIO_2_ > 60% populations, particularly for the mortality rate outcomes in this study. 

## 3. Results

### 3.1. Patient Demographics and Clinical Characteristics

**Figure 1:**
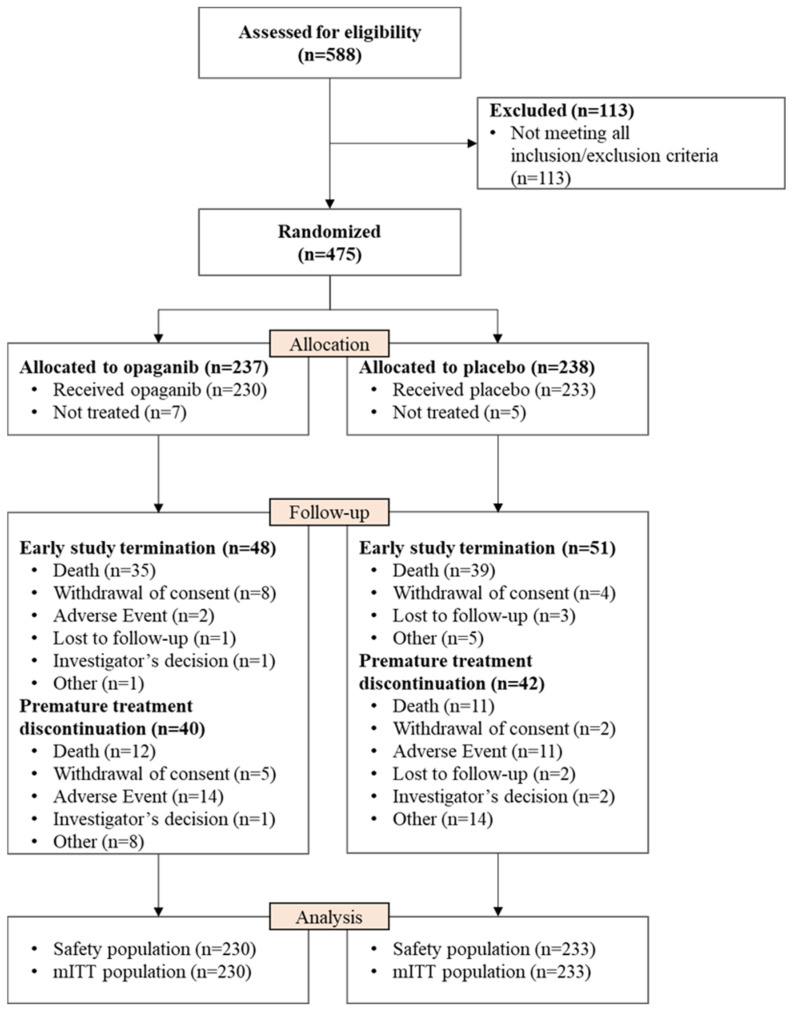
Consort flow diagram. mITT = modified intention to treat, all subjects who were randomized and treated with at least one dose (even partial) of the study drug.

A total of 588 patients were initially screened, and 475 eligible patients were randomized (237 to opaganib and 238 to placebo), as detailed in Figure 1. There were 113 screen failures; the reasons for screen failure are presented in Table S1. Patients randomized but not treated were due to missing inclusion/exclusion information or change in condition that rendered the patient ineligible prior to the first treatment. 

Patient demographics and characteristics were similar between treatment arms, as shown in Table 1. There were no meaningful differences in the parameters measured for demographics. Effective SoC medications for COVID-19 treatments included glucocorticoids (predominantly dexamethasone), remdesivir, and COVID-19 convalescent plasma. Glucocorticoids were the most commonly used, with 94% of all study patients receiving glucocorticoids.

### 3.2. Primary and Secondary Efficacy Outcomes

**Figure 2:**
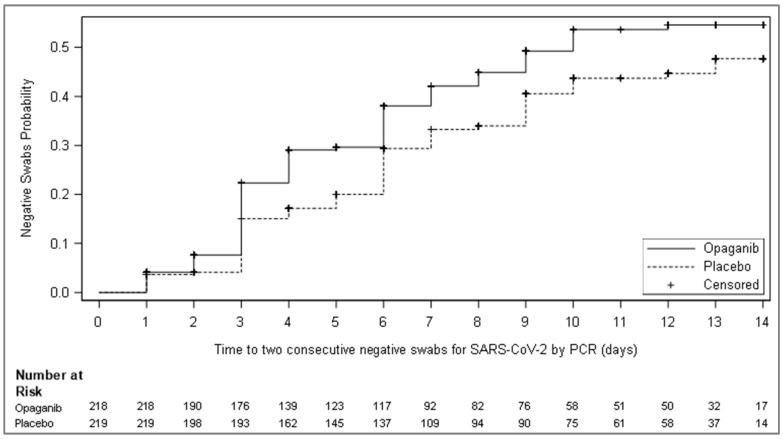
Kaplan–Meier curves of cumulative incidence for time to two consecutive negative swabs for SARS-CoV-2 by RT-PCR, at least 24 h apart, in the mITT population that was RT-PCR positive for SARS-CoV-2 at screening. Twenty-six (26) patients that were excluded from this analysis had an eligibility RT-PCR up to 7 days prior to screening, but not at screening.

While the prespecified primary outcome analysis of the mITT population (any patient who received at least one dose of study drug) was not statistically significant, opaganib was numerically superior to placebo in the mITT population, as shown in Table 2. A consistent, albeit small, numerical benefit was shown across most secondary endpoints (Table S2). For the secondary endpoint of time to viral clearance, despite randomization being a median of 11 days after symptom onset for both opaganib and placebo cohorts, opaganib demonstrated a nominally significant improvement as compared to placebo, with a hazard ratio of 1.34 and a nominal *p*-value of 0.043 as shown in Figure 2 (and Table 3). The median time to viral clearance was 10 days for the opaganib-treated arm compared to >14 days for the placebo arm. Of note, the secondary endpoint requiring serial measurements of temperature could not be evaluated due to the volume of missing data points. 

**Figure 3:**
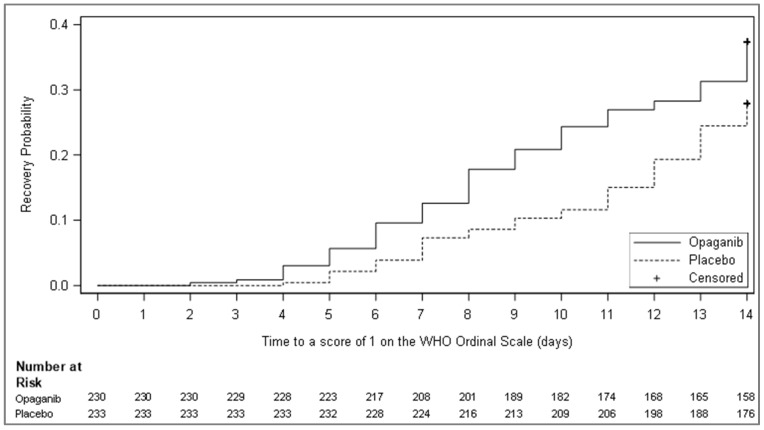
Kaplan–Meier curve of cumulative incidence for time to recovery as defined by improvement to a score of 1 or less on the WHO Ordinal Scale of Clinical Improvement (mITT). Subjects who were lost to follow-up, withdrew consent, or died before day 14 were censored to day 14. Remaining subjects without the event were censored to day 14 or end of study day if it occurred earlier.

Pre-specified strata analyses revealed that mortality was reduced by day 28 (4.7% vs. 21.3%; *p*-value = 0.024) in patients receiving opaganib in addition to remdesivir and corticosteroid SoC (n = 90) and by day 42 (7.0% vs. 23.4%; *p*-value = 0.034; Table 4). Furthermore, time to recovery as defined by improvement to a score of 1 or less on the WHO Ordinal Scale at 14 days of treatment was assessed in the mITT (n = 463) analysis set (Table 5 and Figure 3), as a pre-specified exploratory objective. Opaganib treatment reduced the time until recovery with 86 (37.4%) opaganib-treated patients vs. 65 (27.9%) in the placebo arm recovering by day 14 (*p*-value = 0.013, HR 1.49). 

### 3.3. Post-Hoc Efficacy Analysis 

As mentioned above, for a short period, patients with simple face masks and an oxygen flow rate greater than 5 L/min were allowed due to a lack of adequate high flow devices at several sites. Thus, a pre-specified sensitivity analysis was added during the blinded phase of the protocol that revealed the sub-population comprised of patients treated by either high flow nasal cannula, non-mechanical positive pressure ventilation, or reservoir face masks at baseline (n = 435) demonstrated a reduced treatment benefit with opaganib as shown in Table S3. Thus, we decided to perform two post-hoc analyses of opaganib versus placebo using the other oxygenation parameter that had been collected: one for patients at or below and one for patients above the median value of the FIO_2_ (60%).

Post-hoc analysis of the subpopulation requiring relatively lower FIO_2_ (≤60%; n = 251; Table 6) at baseline suggested an appreciable positive opaganib treatment benefit, with no benefit observed for patients requiring higher FIO_2_ (>60%; n = 193) at baseline. The analysis demonstrated a 21.3% relative increase in patients no longer requiring supplemental oxygen vs. placebo—the primary endpoint (76.9% vs. 63.4%, nominal *p*-value *p* = 0.033). This post-hoc primary endpoint analysis was supported by the outcomes for the secondary endpoints in this subpopulation, associated with clinical outcomes and supplemental oxygen requirements, with nominally significant *p*-values, including for (1) changes in the WHO Ordinal Scale (21% relative increase), (2) the need for intubation/mechanical ventilation (61.8% reduction), and (3) reduction in overall mortality by day 42 (61.8% reduction). 

**Figure 4:**
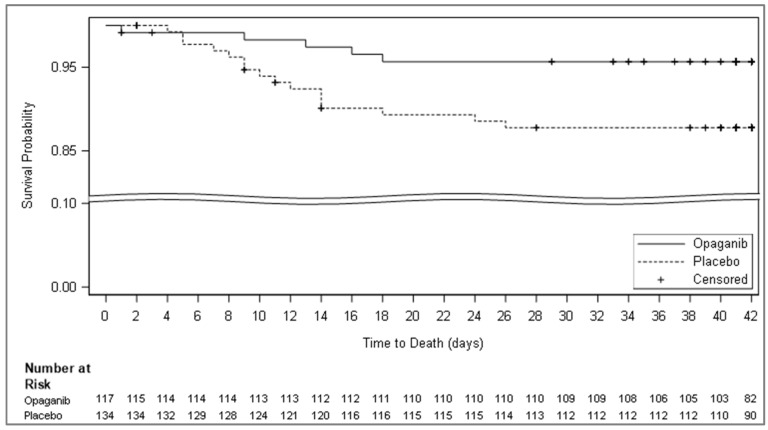
Kaplan–Meier curves of time to death by day 42 (FIO_2_ ≤ 60% population). Kaplan–Meier curve of time to death through the end of day 42 from the mITT population with FIO_2_ ≤ 60% at baseline. Patients were censored at day 42 or at study termination if it occurred before day 42.

A larger proportion of patients in this lower FIO_2_ cohort died in the placebo arm as compared to the opaganib arm (15.67% vs. 5.98% with a % difference of −9.69; 95% CI −17.21, −2.18; *p*-value 0.019; Table 6; Figure 4). Moreover, a larger proportion of patients requiring intubation and ventilation were observed in the placebo arm versus the opaganib arm by day 4 (17.91% vs. 6.84% with % difference of −11.0 7; 95% CI −19.01, −3.13; *p*-value 0.012). While the FIO_2_ is not a standard biomarker, it correlated well with the SpO_2_:FIO_2_ ratio, a validated predictor for ARDS (R = −0.93).

At baseline, lower median lymphocyte counts and higher inflammatory median values were observed in the patients with ≥60% FiO_2_, thus supporting that FIO_2_ positively correlates with disease severity (Table 7). Consistent with the literature, higher LDH, D-dimer, CRP, and ferritin and lower lymphocyte counts were significant risk factors for mortality. Advanced age and oxygen saturation at baseline were additional risk factors that positively correlated with worse outcomes, but higher FIO_2_ was ranked the second highest risk factor for mortality at day 42 in the mITT population (Table 8). No meaningful baseline differences in the values of these biomarkers were detectable when comparing the patients in the opaganib arm to the placebo arm in the low FIO_2_ group (Table 9).

Potential confounder effects on the mortality results derived from the baseline FIO_2_ subpopulation requiring ≤60% supplement oxygen was evaluated via Cox regression analysis (Table S4). We standardized (adjusted) each 42-day survival curve treatment group according to the distribution of respective confounders to potentially identify any effectors. Adjusted mortality rates were in close agreement with crude unadjusted estimates, thus confirming that it is unlikely that confounding effects contributed to the observed effect. In summary, Cox regression analysis of mortality outcomes suggested that the observed effects of opaganib treatment were independent of potential baseline characteristics confounders in this ≤60% FIO_2_ subpopulation. 

### 3.4. Safety Analysis

Of 463 patients in the safety population who received at least one dose of study drug, 67.4% and 63.1% of patients receiving opaganib and placebo, respectively, experienced at least one treatment-emergent adverse event (TEAE). The majority of the TEAEs were mild to moderate in severity. Treatment-emergent serious adverse events (TESAEs) were experienced by 52/230 (22.6%) patients in the opaganib arm versus 52/233 (22.3%) patients in the placebo arm (Table 10 and Table S5). TEAEs with an outcome of death occurred in 36/230 (15.7%) versus 40/233 (17.2%) in the opaganib and placebo arms, respectively. 

Thirty-nine, or 17%, of all adverse events experienced in the opaganib arm were considered related to treatment. In the placebo arm, 29 (12.4%) adverse events were considered related to treatment. Psychiatric adverse events included insomnia, which occurred at a frequency of greater than 5% (7.4% versus 3.9% in the opaganib and placebo arm, respectively). Insomnia is included as an AE of special interest (AESI) and discussed with other neuropsychiatric events below. The only TESAE deemed to be related to drug treatment by the investigators while still blinded was a single incident of grade 2 change in mental status that resolved within 24 h of the medication being withdrawn. 

Premature discontinuation from the study (between days 15 and 42, inclusively) occurred in 48 (20.9%) and 51 (21.9%) patients in the opaganib and placebo arms, respectively. Overall, there were 76 treatment-emergent deaths in the study (Table 10). Most of the deaths in both groups were assessed as related to COVID-19 and associated complications. No deaths were assessed as being related to the study drug.

Due to high mortality in a blinded review, RedHill suspended enrolment at site 114 in October 2020, as discussed above (see also Table S6). 

## 4. Discussion

This was a phase 2/3 multi-center randomized, double-blind, parallel arm, placebo-controlled study in 475 adult patients hospitalized with severe SARS-CoV-2 (COVID-19) pneumonia evaluating the treatment benefit of opaganib therapy vs. placebo. Primary analysis was based on the proportion of patients who no longer needed supplemental oxygen by day 14 (end of treatment), with secondary objectives looking at improvement in other clinical outcomes such as mortality. 

As an SK2 selective inhibitor [5], opaganib is unique in its mechanism of action and, to our knowledge, is the only sphingosine kinase inhibitor that is under investigation for the treatment of viral diseases, including COVID-19.

The pre-specified analyses in the mITT showed no significant differences with treatment, with the nominal exceptions of shorter time to viral clearance, superior outcomes for patients receiving SoC of both remdesivir and dexamethasone in addition to opaganib, and improved time to recovery to WHO level 1 in patients treated with opaganib. A post-hoc analysis of the data, utilizing oxygen requirements at baseline to refine the categorization of COVID-19 pneumonia severity, further justified by differences in baseline inflammatory markers and lymphocyte counts, suggested that in a subpopulation of patients requiring FIO_2_ (≤60%) at baseline, patients receiving opaganib had better outcomes for both primary and secondary endpoints compared to patients receiving placebo. Overall, the safety events were similar between treatment arms. 

The cutoff value of 60% was chosen as it represented the median FIO_2_ of the mITT population, rather than a data-driven cutoff based on the optimal FIO_2_ observed in this protocol. Importantly, oxygen requirement as measured by baseline FIO_2_ emerged as a novel potential metric for prediction of treatment benefit in severely hospitalized patients requiring supplemental oxygen regardless of oxygen delivery device that correlated well with the SpO_2_:FIO_2_ ratio. Post-hoc analyses of this subpopulation revealed that patients receiving opaganib had nominally superior outcomes across the primary and secondary measures, including a 61.8% reduction in mortality by day 42, over patients receiving placebo. This novel metric for refining categorization of severity within the WHO level 5 population is supported by analyses of biomarkers; the difference in outcomes defined by FIO_2_ requirement is correlated with baseline prognostic lymphocyte counts, inflammatory markers, and D-dimer levels. These data support FIO_2_ as a baseline predictor of treatment benefit within this patient population.

Considering that baseline FIO_2_ was the second highest risk factor for mortality, these data suggest that opaganib may be effective in reducing the incidence of mortality in hospitalized patients with severe COVID-19 pneumonia as indicated by FIO_2_ requirements up to and including 60%.

By contrast, in the subpopulation of patients requiring FIO_2_ > 60% at baseline, a difference in outcomes was not observed, suggesting that the overall limited effect seen in the mITT population could be attributed to this cohort of patients. The lack of treatment effect in this subpopulation may be explained by the greater severity of the underlying COVID-19 lung disease reflected by the higher inspired oxygen requirements, lower lymphocyte counts, and elevated inflammatory markers at baseline, which may, in turn, suggest that there may be a threshold level for disease irreversibility [13].

Cox regression analysis demonstrated that mortality outcome differences in the FIO_2_ ≤ 60% subpopulation between opaganib and placebo were independent of potential baseline characteristics confounders; in addition, the differences in severity between the low FIO_2_ (≤60%) and high FIO_2_ (>60%) subpopulations were independent of disease duration.

As illustrated by this global study, current severity classifications by the WHO Ordinal Scale for Clinical Improvement are rather general and may not be homogeneous for a diversity of settings across global sites. As a result, the type of oxygen delivery device may not be sufficient to define COVID-19 pneumonia severity. As the objective of supplemental oxygen is to deliver sufficient FIO_2_ to the patient, it stands to reason that the FIO_2_ requirement at baseline may serve as a better proxy for refining how disease severity is measured. 

Overall, oral administration of opaganib treatment in this large-controlled study was shown to be relatively safe and well-tolerated. The safety profile did not indicate any new safety concerns with respect to the use of opaganib in this hospitalized patient population requiring supplemental oxygen for COVID-19 pneumonia. Generally, gastrointestinal (primarily low-grade nausea), and neuropsychiatric disorders (primarily low-grade insomnia) occurred more frequently in the opaganib arm, while respiratory disorders occurred more frequently in the placebo arm. These safety results may reflect the expected opaganib adverse events for the opaganib arm, while reflecting disease progression of COVID-19 pneumonia for the placebo arm. Except for neuropsychiatric events, TEAEs of special interest were similar between treatment arms. These infrequent neuropsychiatric events occurred more commonly in patients in the opaganib arm and were mostly of mild severity. 

Currently, there are limited treatment options for patients with severe COVID-19 pneumonia [14]. The emergence of new variant strains diminishes the effectiveness of both antibodies and vaccines. While the IL6 inhibitors, like tocilizumab, were positive in just one positive trial to achieve EUA approval, they were less effective for severe patients [15,16,17]. Molnupiravir and paxlovid show varying degrees of efficacy in outpatients within 5 days of symptom onset [18,19,20]. In our study, severely ill patients were randomized a median of 11 days after the appearance of symptoms. Additionally, the viral clearance data demonstrate further support for opaganib’s antiviral activity. Importantly, by addressing a far more advanced disease status and a mechanism that is host-based and should be agnostic to viral variants, opaganib has the potential to fill an urgent unmet medical need for hospitalized patients with COVID-19 without effective treatment options currently available. 

This study has two major limitations: First, the positive data arising from post-hoc analysis of patients with median FIO_2_ or less should be repeated in a prospective study. Second, while all enrolled patients were affected by COVID-19, the results may not fully be generalizable to all types of patient co-morbidities due to populations excluded by eligibility criteria.

## 5. Conclusions

While the prespecified primary endpoint was not statistically significant, a post-hoc analysis suggested a potential treatment benefit in the subpopulation of hospitalized patients with severe COVID-19 pneumonia as defined by WHO 5 criteria, requiring an FIO_2_ of ≤60%. This finding was supported by lower inflammatory markers and higher lymphocyte counts at baseline. The safety profile was favorable, indicating a favorable overall risk–benefit for the treatment of COVID-19. These data, combined with a shorter time to viral clearance, indicate that opaganib may be an effective new oral therapy for COVID-19 and that the baseline FIO_2_ requirement may be a new clinical biomarker for patient selection. Further prospective studies are warranted to prospectively confirm the benefit of opaganib treatment for this subpopulation of WHO 5 patients as well as studies of opaganib in less severe patients with COVID-19.

## Figures and Tables

**Table 1:** Subject demographics and baseline characteristics (mITT).

	No. (%)		
Parameter	Opaganib (N = 230)	Placebo (N = 233)	Overall (N = 463)
Age (years)	55.7 (28–80)	57.3 (26–80)	56.5 (26–80)
Sex			
Male	145 (63.0)	158 (67.8)	303 (65.4)
Female	85 (37.0)	75 (32.2)	160 (34.6)
Ethnicity			
Hispanic or Latino	68 (29.6)	65 (27.9)	133 (28.7)
Not Hispanic or Latino	158 (68.7)	162 (69.5)	320 (69.1)
Unknown	4 (1.7)	6 (2.6)	10 (2.2)
Race			
White	192 (83.5)	198 (85.0)	390 (84.2)
American Indian or Alaska Native	5 (2.2)	4 (1.7)	9 (1.9)
Asian	1 (0.4)	0	1 (0.2)
Black or African American	10 (4.3)	10. (4.3)	20 (4.3)
Native Hawaiian or Other Pacific Islander	0	0	0
White/Black or African American	1 (0.4)	2 (0.9)	3 (0.6)
White/American Indian or Alaska Native	0	1(0.4)	1 (0.2)
Other	21 (9.1)	18 (7.7)	38 (8.4
Smoking status			
Never	169 (73.5)	169 (72.5)	338 (73.0)
Former	35 (15.2)	32 (13.7)	67 (14.5)
Current	11 (4.8)	15 (6.4)	26 (5.6)
Missing	15 (6.5)	17 (7.3)	32 (6.9)
HbA1c at screening			
<6.5 and not on active treatment with insulin or oral hypoglycemics	147 (63.9)	153 (65.7)	300 (64.8)
≥6.5 or on active treatment with insulin or oral hypoglycemics	83 (36.1)	79 (33.9)	162 (35.0)
Missing	0	1 (0.4)	1 (0.2)
BMI at baseline (Kg/m^2^), n =	223	228	451
Mean (SD)	31.02 (5.676)	30.47 (5.158)	30.74 (5.421)
Median (min, max)	30.39 (20.1, 54.8)	29.73 (19.6, 49.4)	29.99 (19.6, 54.8)
Supplemental oxygen at baseline			
Yes	229 (99.6)	233 (100)	462 (99.8)
No	1 (0.4)	0	1 (0.2)
Oxygen type (for oxygen requirement—yes) *			
Intubation/mechanical ventilation	1 (0.4)	2 (0.9)	3 (0.6)
Low flow nasal cannulas	3 (1.3)	1 (0.4)	4 (0.9)
Non-invasive ventilation/HFNC/mask with reservoir/mask without reservoir	225 (98.3)	230 (98.7)	455 (98.5)
type of non-invasive ventilation/HFNC/mask with reservoir/mask without reservoir			
BIPAP	5 (2.2)	7 (3.0)	12 (2.6)
CPAP	64 (27.9)	61 (26.2)	125 (27.1)
HFNC	82 (35.8)	88 (37.8)	170 (36.8)
Face mask (without reservoir)	9 (3.9)	11 (4.7)	20 (4.3)
Non-rebreather (reservoir) mask =	65 (28.4)	63 (27.0)	128 (27.7)
Not on non-invasive ventilation/HFNC/mask with reservoir/mask without reservoir	4 (1.7)	3 (1.3)	7 (1.5)
Oxygen flow at baseline, L/min, n = , mean (SD) ^†^	222 24.6 (19.59)	219 24.9 (19.83)	441 24.8 (19.69)
Oxygen in the gas mix (%), n = , mean (SD)	222 65.5 (18.31)	222 64.1 (19.00)	444 64.8 (18.65)
Temperature at baseline (Celsius), n = mean (SD)	226 36.79 (0.641)	232 36.78 (0.586)	458 36.78 (0.613)
Respiratory rate at baseline (breaths/minute), n = , mean (SD)	198 21.9 (4.86)	205 21.7 (4.78)	403 21.8 (4.81)
Oxygen saturation at baseline (%), n = , mean (SD) ^‡^	229 93.2 (5.03)	233 93.0 (5.04)	462 93.1 (5.03)
Systolic blood pressure at baseline (mmHg), n = , mean (SD)	229 127.0 (15.04)	233 127.9 (15.12)	462 127.5 (15.07)
Diastolic blood pressure at baseline (mmHg), n = , mean (SD)	229 75.7 (10.32)	233 75.9 (10.51)	462 75.8 (10.40)
Pulse rate at baseline (beats/min), n = , mean (SD)	229 78.6 (12.70)	233 78.6 (13.08)	462 78.6 (12.88)
Efficacious SoC concomitant medication ^§^			
Glucocorticoids	217 (94.3)	219 (94.0)	
Remdesivir	43 (18.7)	37 (15.9)	
Hyperimmune plasma COVID-19	3 (1.3)	5 (2.1)	
COVID-19 vaccine	0	1 (0.4)	

All baseline oxygen variables are taken from the last observation prior to exposure or if time is not available from the exposure date. * Percentages are calculated from patients who required oxygen at baseline. ^†^ Baseline oxygen flow was only collected among patients not intubated at baseline who required oxygen. ^‡^ Patients who could not be taken off of oxygen to measure their saturation on room air (essentially all) had measurements taken while on supplemental oxygen. ^§^ Concomitant medications received at any timepoint during the treatment period of 14 days.

**Table 2:** Primary endpoint analysis: proportion of subjects no longer requiring supplemental oxygen for at least 24 h by day 14 (mITT).

	No. (%)		
Parameter	Opaganib (N = 230)	Placebo (N = 233)	Opaganib vs. Placebo
Patients no longer receiving supplemental Oxygen (“Success” *), n (%)	139 (60.43)	132 (56.65)	
Difference ^†^			+3.78
95% CI for the difference ^†^			−5.19, 12.75
*p*-value ^†^			0.391
“Failure” ^‡^	91 (39.57)	101 (43.35)	
Due to the need for supplemental oxygen at day 14, n (%)	70 (30.43)	75 (32.19)	
Due to death up to day 14, n (%)	18 (7.83)	21 (9.01)	
Due to lost to follow-up by day 14, n (%)	3 (1.30)	4 (1.72)	
Due to missing status at day 42 with prior success, n (%)	0	1 (0.43)	

* Success indicates that a patient was no longer receiving supplemental oxygen for at least 24 h by day 14. ^†^ *p*-value from the Cochran–Mantel–Haenzel test using the study stratification factors used for randomization and corresponding stratified proportion difference with 95% CI. All reported *p*-values are two-sided. Patients can only be in one failure category. ^‡^ Patients who die within 42 days or are LTFU or in need of oxygen up to 42 days are regarded as failing.

**Table 3:** The time to two consecutive negative swabs for SARS-CoV-2 by RT-PCR, at least 24 h apart, up to 14 days.

Parameter	Opaganib (N = 218)	Placebo (N = 219)
The time to two consecutive negative swabs for SARS-CoV-2 by RT-PCR, at least 24 h apart, up to 14 days		
Number of events	93 (42.7)	79 (36.1)
Number of censored observations	125 (57.3)	140 (63.9)
Reasons for censoring		
No post-baseline results available	13	8
Less than two results and discharged by day 5	7	7
Less than two results and not discharged by day 5	21	24
At least two results that are not two sequential negatives	84	101
Log-rank test statistic *	12.58	
*p*-value *	0.043	
Hazard ratio (HR) and 95% CI ^†^	1.34 (0.99–1.82)	
Kaplan–Meier median estimate and 95% CI ^‡^	10.00 (8.00–NA)	NA (10.00–NA)
Cumulative incidence {%} ^‡^		
Day 7	42.09	33.25
95% CI	35.19, 49.75	26.89, 40.65
Day 14	54.57	47.69
95% CI	46.87, 62.63	39.70, 56.41

* Analysis statistics were estimated using a log-rank test stratified by study stratification factors used for randomization. A negative (positive) statistic is associated with longer (shorter) time to the event. ^†^ Estimates and confidence intervals are obtained from the Cox proportional hazards regression model with treatment group as an explanatory variable and stratification factors to determine the strata levels. ^‡^ Estimated using the Kaplan–Meier estimator. NA = Not applicable due to fewer than 50% of the group having reached the necessary event.

**Table 4:** Mortality due to any cause at days 28 and 42 after remdesivir and corticosteroids, with or without (placebo) opaganib for the mITT population.

	No. (%)		
Parameter	Opaganib	Placebo	Outcome
Mortality due to any cause at day 28 *	2 (4.65)	10 (21.28)	
Difference (Opaganib% − Placebo%)			−16.63
Percentage change (Opaganib%/placebo% × 100)			−81.1
95% CI	0.00, 10.95	9.58, 32.98	−29.91, −3.34
*p*-value			0.024
Mortality due to any cause at day 42	3 (6.98)	11 (23.40)	
Percentage change (Opaganib%/placebo% × 100)			−16.43
95% CI	0.00, 14.59	11.30, 35.51	−30.73, −2.1
*p*-value			0.034

* Mortality (“failure”) is assessed by treatment day 28 or 42 (including). Any early termination/missing survival status at the EOS visit is also regarded as failure for the primary analysis of this endpoint.

**Table 5:** Time to recovery as defined by improvement to a score of 1 or less on the WHO Ordinal Scale for Clinical Improvement in the mITT population.

Parameter	Opaganib (N = 230)	Placebo (N = 233)
Time to recovery as defined by improvement to a score of 1 or less on the WHO Ordinal Scale for Clinical Improvement, up to 14 days		
Number of events (%)	86 (37.4)	65 (27.9)
Number of censored observations (%)	144 (62.6)	168 (72.1)
Log-rank test statistic ^1^	14.89	
*p*-value ^1^	0.013	
Hazard ratio (HR) and 95% CI ^2^	1.49 (1.08–2.05)	
Kaplan–Meier median estimate and 95% CI ^3^	NA (NA–NA)	NA (NA–NA)
Cumulative incidence, % ^3^		
Day 7	12.61	7.30
Day 14	37.39	27.90

CI = confidence interval; HR = hazard ratio; NA = not achieved. ^1^ Analysis statistics were estimated using a log-rank test stratified by study stratification factors used for randomization. A negative (positive) statistic is associated with longer (shorter) time to the event. ^2^ Estimates and confidence intervals were obtained from the Cox proportional hazards regression model with treatment group as an explanatory variable and stratification factors as covariates. ^3^ Estimated using the Kaplan–Meier estimator. NA = Not applicable due to fewer than 50% of the group having reached the necessary event.

**Table 6:** Post-hoc primary and secondary outcomes in patients with a baseline FIO_2_ of <60%.

	No. (%)		
	Opaganib (N = 117)	Placebo (N = 134)	Opaganib vs. Placebo
**Primary Outcomes**			
Patients no longer receiving supplemental oxygen (“Success”) *	90 (76.9)	85 (63.4)	
Difference ^†^			13.49
95% CI for the difference ^†^			2.32, 24.66
*p*-value ^†^			0.033
“Failure”	27 (23.08)	49 (36.57)	
Due to the need for supplemental oxygen at day 14	24 (20.51)	32 (23.88)	
Due to death up to day 14	3 (2.56)	13 (9.70)	
Due to lost to follow-up by day 14	0	3 (2.24)	
Due to missing status at day 42 with prior success	0	1 (0.75)	
**Secondary Outcomes**			
Patients with an improvement of 2 or more on the WHO Ordinal Scale compared to baseline by day 14 ^‡^	93 (79.49)	88 (65.67)	
Difference			13.82
Percentage change (Opaganib%/Placebo% × 100^−1^)			21
*p*-value			0.023
Time to a score of ≤3 on the WHO Ordinal Scale			
Number of events	93 (79.5)	88 (65.7)	
Kaplan–Meier median (days)	8.00	10.00	
95% CI	7.00–9.00	9.00–12.00	
*p*-value			0.010
Time to low oxygen flow via nasal cannula (from high flow nasal cannula or CPAP/BiPAP)			
Number of events	102 (87.2)	106 (79.1)	
Kaplan–Meier median (days)	4.00	5.00	
95% CI	3.00–5.00	4.00–6.00	
*p*-value			0.028
Time to discharge by day 42			
Number of Events (%)	110 (94.0)	109 (81.3)	
Kaplan–Meier median (days)	10.00	14.00	
95% CI	9.00–13.00	11.00–14.00	
*p*-value ^§^			0.004
Patients requiring intubation and mechanical ventilation by day 42 ^ll^	8 (6.84)	24 (17.91)	
Difference			−11.07
Percentage change (Opaganib%/Placebo% × 100^−1^)			−61.8
95% CI	2.26, 11.41	11.42, 24.40	−19.01, −3.13
Intubation without death	2 (1.71)	3 (2.24)	
Intubation with death	4 (3.42)	13 (9.70)	
Death without intubation	1 (0.85)	3 (2.24)	
Early termination/missing data (alive without intubation)	1 (0.85)	5 (3.73)	
*p*-value			0.012
Mortality due to any cause at day 42 (“Failure”) **	7 (5.98)	21 (15.67)	
Difference			−9.69
Percentage change (Opaganib%/Placebo% × 100^−1^)			−61.8
95% CI	1.69, 10.28	9.52, 21.83	−17.20, −2.18
*p*-value			0.019

N (and n) = number; CI = confidence interval. * Success indicating that a patient no longer received supplemental oxygen for at least 24 h by day 14. ^†^ Nominal *p*-value from the Cochran–Mantel–Haenszel test using the study stratification factors used for randomization and corresponding stratified proportion difference with 95% CI. All reported *p*-values are nominal and two-sided. Patients can only be in one failure category. ^‡^ Success was defined as a subject who reached improvement of at least two points on the WHO Ordinal Scale by day 14, maintained by the end of the study (EOS). ^§^ Estimated using the Kaplan–Meier estimator. The standard error of the medinas was estimated using the bootstrap method with a 10,000 sample size. ^ll^ Patients who died within 42 days or were LTFU or in need of oxygen up to 42 days have been regarded as failing. For secondary outcomes, failure was defined as any requirement of intubation and mechanical ventilation or death without intubation by day 42. Early termination of study (or failure to complete EOS visit) is also considered failure. ** Mortality (“failure”) is assessed by treatment day 42 (including). The same results were demonstrated for day 28. Any early termination/missing survival status at the EOS visit is also regarded as failure for the primary analysis of this endpoint.

**Table 7:** Biomarker medians by subpopulations (FIO_2_ ≤ 60% and FIO_2_ > 60% at baseline).

	Low FIO_2_			High FIO_2_			
Marker	N	Median	Q1, Q3	N	Median	Q1, Q3	*p*-Value *
Lymphocytes (10^9^/L)	249	0.990	0.71, 1.38	178	0.780	0.50, 1.20	<0.0001
C reactive protein (mg/L)	240	60.800	21.40, 153.40	186	102.800	38.67, 200.30	0.0005
Ferritin (µg/L)	228	666.950	370.17, 1297.50	167	1000.000	500.60, 2000.00	0.0008
D-dimer (µg/mL)	240	0.450	0.18, 1.06	178	0.806	0.31, 1.79	0.0004
Lactate dehydrogenase (IU/L)	230	361.150	290.60, 522.00	183	469.230	344.00, 644.00	<0.0001

* Comparing FIO_2_ ≤ 60% vs. FIO_2_ > 60% subpopulations for imbalances.

**Table 8:** Baseline risk factors for mortality (mITT population).

			Mortality at 42-Day-Rate (%) (Kaplan–Meier Analysis)				
Variable	# Subjects with bl. Data	Cut-Point for Low High Groups (Median)	High Marker Group	Low Marker Group	High–Low Difference	CI	Two-Sided *p*-Value
Age	463	57.000	26.2%	7.0%	19.2%	[12.5%, 25.8%]	<0.0001
Oxygen in gas mix at baseline (%)	463	60.000	27.5%	8.5%	18.9%	[11.6%, 26.2%]	<0.0001
# of risk factors	463	3.000	25.5%	8.7%	16.8%	[9.9%, 23.7%]	<0.0001
Lactate dehydrogenase (IU/L)	431	405.000	24.5%	8.6%	16.0%	[9.0%, 22.9%]	<0.0001
Lymphocytes (10^9^/L)	457	0.900	9.1%	23.7%	−14.6%	[−21.4%, −7.8%]	<0.0001
D-dimer (µg/mL)	436	0.578	21.5%	11.0%	10.6%	[3.6%, 17.5%]	0.0029
C reactive protein (mg/L)	445	82.800	21.9%	11.5%	10.4%	[3.4%, 17.4%]	0.0036
Ferritin (µ/L)	411	758.000	21.5%	11.6%	9.9%	[2.6%, 17.1%]	0.0075
Oxygen saturation at baseline (%)	463	94.000	11.8%	20.1%	−8.3%	[−15.0%, −1.6%]	0.0152
Pulse rate at baseline (beats/min)	463	79.000	19.6%	13.7%	6.0%	[−0.9%, 12.9%]	0.0903
BMI (CRF) at baseline (kg/m^2^)	463	29.988	18.9%	14.1%	4.8%	[−2.1%, 11.7%]	0.1754
Systolic blood pressure at baseline (mmHg)	463	129.000	18.9%	14.2%	4.7%	[−2.1%, 11.6%]	0.1770
Weight at baseline (kg)	463	87.100	17.3%	15.3%	2.0%	[−4.9%, 8.9%]	0.5693
Oxygen flow at baseline (L/min)	463	15.000	17.9%	16.1%	1.8%	[−5.5%, 9.0%]	0.6274
Time from the onset of symptoms to randomization (Days)	455	11.000	17.0%	15.8%	1.3%	[−5.7%, 8.3%]	0.7200
Temperature at baseline (C)	463	36.700	17.3%	16.1%	1.2%	[−5.8%, 8.1%]	0.7409

**Table 9:** Biomarker distribution (medians and 1st and 3rd quartiles) by treatment arm (FIO_2_ ≤ 60% subpopulation).

	Opaganib			Placebo			
Biomarker	N	Median	Q1, Q3	N	Median	Q1, Q3	*p*-Value *
Lymphocytes (10^9^/L)	116	0.910	0.69, 1.36	133	1.010	0.72, 1.41	0.2534
C Reactive Protein (mg/L)	113	67.100	26.77, 173.00	127	45.000	18.60, 145.60	0.1910
Ferritin (µg/L)	104	727.600	381.65, 1383.45	124	592.300	368.02, 1201.20	0.3025
D-Dimer (µg/mL)	111	0.499	0.17, 1.17	129	0.380	0.18, 1.04	0.6619
Lactate Dehydrogenase (IU/L)	106	359.400	300.00, 507.00	124	368.750	287.55, 536.34	0.5522

* Comparing the opaganib vs. placebo arms for imbalances. Baseline troponin was collected in 20% of the patients and therefore is not presented.

**Table 10:** Summary of treatment-emergent adverse events by treatment group (safety population).

	No. (%)	
Event	Opaganib * (N = 230)	Placebo * (N = 233)
Any TEAEs	155 (67.4)	147 (63.1)
Serious TEAEs	52 (22.6)	52 (22.3)
Grade 1 TEAEs mild	115 (50.0)	109 (46.8)
Grade 2 TEAEs moderate	58 (25.2)	67 (28.8)
Grade 3 TEAEs severe	32 (13.9)	29 (12.4)
Grade 4 TEAEs life-threatening	17 (7.4)	16 (6.9)
Grade 5 TEAEs death	36 (15.7)	40 (17.2)
Treatment-related TEAEs	39 (17.0)	29 (12.4)
Treatment-related TESAEs	1 (0.4) ^†^	0
TEAEs resulting in dose reduced	2 (0.9)	3 (1.3)
TEAEs resulting in drug withdrawn	26 (11.3)	26 (11.2)

* Patients are counted only once in each system organ class category and only once in each preferred term category. ^†^ Grade 2 event that resolved within 24 h of study drug cessation.

## Data Availability

Data are contained within the article and Supplementary Materials.

## Supplementary Materials

The following supporting information can be downloaded at: https://www.mdpi.com/article/10.3390/microorganisms12091767/s1, Table S1: Reasons for Screen Failures; Table S2: Secondary Outcomes for mITT Population; Table S3: Sensitivity Analysis of the Primary Endpoint: Percentage of Patients No Longer Requiring Supplemental Oxygen, for at Least 24 Hours by Study Day 14; Table S4: Mortality Sensitivity Analysis for Handling Early Study Discontinuation 42 Days for mITT with Baseline FIO2 ≤ 60% Adjusted for Potential Baseline Confounder; Table S5: TESAEs by System Organ Class and Preferred Term (Safety Population); Table S6: TEAES with an Outcome of Death, SoC and Preferred Term by Treatment Group with and without Site 114.
