# Supplementary material for: Effect of Opaganib on Supplemental Oxygen and Mortality in Patients with Severe SARS-CoV-2 Based upon FIO2 Requirements
Source: Microorganisms. 2024 Aug 26;12(9):1767. doi: 10.3390/microorganisms12091767 (PMC11434591; doi:10.3390/microorganisms12091767)
Supplement: Supplementary file 1 [file microorganisms-12-01767-s001.zip › microorganisms-3077704-supplementary.pdf]

Effect of Opaganib on Supplemental Oxygen and Mortality in Patients with Severe SARS-CoV-2 Based Upon FIO_2 Requirements

Supplementary Tables

Table S1. Reasons for Screen Failures.

Unmet Eligibility Criteria	n (%) *
Abnormal Liver Function Tests	53 (46.9)
Prohibited con-meds	45 (38.9)
Negative COVID-19 nasopharyngeal swab	37 (32.7)
Screening QTC above eligibility criteria	31 (27.4)
Intubation at baseline	30 (26.6)

*Patients may have more than one exclusion criteria to fail eligibility.

Table S2. Secondary Outcomes for mITT Population.

	No. (%)		
**Parameter**	**Opaganib**	**Placebo**	**Outcome**
**Patients with an Improvement of 2 or More on the WHO Ordinal Scale Compared to Baseline by Day 14, n (%)***	145 (63.04)	138 (59.23)	
Difference (Opaganib% - Placebo%)			3.81
Percentage change (Opaganib%/placebo%*100)			+6.4
95% CI	56.81, 69.28	52.92, 65.54	-5.06, 12.69
**Time to a score of ≤ 3 on the WHO Ordinal Scale, days**			
Number of events, n (%)	145 (63.0)	138 (59.2)	
Stratified Log-Rank Test, p-value †			0.231
Kaplan-Meier Median (days) ‡	11.00	12.00	
Estimate and 95% CI	9.00 - 13.00	10.00 - 14.00	
**Time to low oxygen flow via nasal cannula (from high flow nasal cannula or CPAP/BiPAP), days**			
Number of events, n (%)	169 (73.5)	167 (71.7)	
Stratified Log-Rank Test, p-value			0.472
Kaplan-Meier Median (days), Estimate 95% CI	5.50 (5.00 - 6.00)	6.00 (5.00 - 7.00)	
**Time to Discharge by day 14**			
Number of Events n (%)	128 (55.7)	132 (56.7)	
Stratified Log-Rank Test, p-value			0.521
Kaplan-Meier Median (days)	13.50	14.00	
95% CI	12.00 - 15.00	NA – NA	
**Patient requiring intubation and mechanical ventilation by day 42[d]**	53 (23.04)	57 (24.46)	
Difference (Opaganib% - Placebo%)			-1.42
Percentage change (Opaganib%/placebo%*100-1)			-5.8
95% CI	17.60, 28.49	18.94, 29.98	-9.17, 6.33
Intubation Without death, n (%)	10 (4.35)	10 (4.29)	
Intubation with Death, n (%)	27 (11.74)	30 (12.88)	
Death without intubation, n (%)	9 (3.91)	8 (3.43)	
Early termination/missing data (alive without intubation), n (%)	7 (3.04)	9 (3.86)	
Difference in the proportion of "Failure" between groups §			0.701
Stratified proportion difference ll			-1.51

95% CI			-9.19, 6.18
Difference in Rates (Opaganib - Placebo)			-2.36
95% CI	11.72, 21.32	13.86, 23.91	-9.31, 4.59
Difference in the proportion of "Failure" between groups **			0.488
**Mortality due to any cause at day 42 (“Failure”), n (%)**	44 (19.13)	47 (20.17)	
Difference (Opaganib% - Placebo%)			-1.04
Percentage change (Opaganib%/placebo%*100-1)			-5.2%
95% CI	14.05, 24.21	15.02, 25.32	-8.28, 6.20
Difference in the proportion of "Failure" between groups			0.755

*Success definition: patient who reached improvement of at least 2 points on WHO scale by Day 14 and maintained this by end of study (EOS). [†]p-value from Cochran Mantel-Haenzel test using the study stratification factors used for randomization, and corresponding stratified proportion difference with 95% CI. [‡]Estimated using the Kaplan-Meier estimator. [§]Failure definition: any requirement of intubation and mechanical ventilation, or death without intubation, by day 42. Early termination of study (or failure to complete EOS visit) is also considered as failure. [‖]A Cochran Mantel-Haenszel test to compare the proportion of failure between the two groups including stratified proportion difference and its 95% CI, using the study stratification factors used for randomization **Mortality (one of the reasons that define "failure") is assessed up to and including Day 42. Any early termination/missing survival status at EOS visit is also regarded as “failure” for the primary analysis of this endpoint.

Table S3. Sensitivity Analysis of the Primary Endpoint: Percentage of Patients No Longer Requiring Supplemental Oxygen, for at Least 24 Hours by Study Day 14.

**BLHF Treated Population** *	**Statistics**	**Opaganib (N=216)**	**Placebo (N=219)**	**Outcome**
**Patients No Longer Receiving Supplemental Oxygen (“Success”)** [†]	n (%)	126 (58.33)	125 (57.08)	
Difference in Rates (Opaganib - Placebo)	%			1.26
	95% CI	51.76, 64.91	50.52, 63.63	-8.03, 10.54
	p-value [2]			0.755
Stratified Success Proportion Difference (CI) [‡]	% (CI)			1.45 (-7.64, 10.55)
“Failure” [§]	n (%)	90 (41.67)	94 (42.92)	
Due to need of supplemental oxygen at Day 14	n (%)	70 (32.41)	72 (32.88)	
Due to death up to day 14	n (%)	18 (8.33)	19 (8.68)	
Due to Lost to follow up by Day 14	n (%)	2 (0.93)	2 (0.91)	
Due to missing status at Day 42 with prior success	n (%)	0	1 (0.46)	

N (and n) =number; CI = Confidence Interval. * ”Baseline high flow” patients defined as receiving supplemental oxygenation at baseline either by non-rebreather (reservoir) face mask, high flow nasal cannulas or non-invasive positive pressure ventilation. [†]Success indicating that a patient no longer received

supplemental oxygen for at least 24 hours by Day 14. ‡p-value from Cochran Mantel-Haenzel test using the study stratification factors used for randomization, and corresponding stratified proportion difference with 95% CI. All reported p-values are nominal, two sided. Patients can only be on one failure category. §Patients who died within 42-days or were LTFU or in need of oxygen up to 42 days have been regarded as Failure.

Table S4. Mortality Sensitivity Analysis for Handling Early Study Discontinuation 42 Days for miTT with Baseline FIO2≤60% Adjusted for Potential Baseline Confounder.

	**Adjusted Mortality at 42 days Analysis**				
	**Mortality rates**		**Opaganib-Placebo difference**		
**Potential confounder**	**Opaganib**	**Placebo**	**Difference**	**lower 95% CI for difference**	**upper 95% CI for difference**
Study Site Identifier	4.6%	10.9%	-6.3%	-12.5%	-0.1%
# of risk factors :<=/> overall median	4.7%	11.2%	-6.4%	-12.9%	-0.0%
# of risk factors	4.6%	11.2%	-6.6%	-12.9%	-0.3%
Age :<=/> overall median	4.6%	11.5%	-6.9%	-13.4%	-0.4%
Age	4.5%	11.7%	-7.2%	-13.7%	-0.6%
Pulse Rate at Baseline (beats/min) :<=/> overall median	4.5%	11.7%	-7.2%	-13.7%	-0.7%
Cardiovascular Disease (Curr)	4.4%	11.7%	-7.3%	-13.7%	-0.8%
Male (Curr)	4.5%	11.9%	-7.4%	-14.2%	-0.7%
Positive for COVID at Screening	4.4%	11.9%	-7.5%	-14.1%	-0.8%
Time from onset of symptoms to randomization (Days) :<=/> overall median	4.5%	12.1%	-7.7%	-14.4%	-0.9%
Renal Disease (Curr)	4.3%	12.1%	-7.8%	-14.4%	-1.1%
Smoking Status	4.7%	12.4%	-7.8%	-14.8%	-0.7%
Oxygen Saturation at Baseline (%) :<=/> overall median	4.4%	12.1%	-7.8%	-14.5%	-1.1%
Weight at Baseline (kg) :<=/> overall median	4.4%	12.2%	-7.8%	-14.5%	-1.0%
Three or More Risk Factors (Curr)	4.3%	12.1%	-7.8%	-14.3%	-1.3%
Country	4.3%	12.1%	-7.8%	-14.5%	-1.2%
Oxygen in Gas Mix at Baseline (%) :<=/> overall median	4.3%	12.2%	-7.8%	-14.6%	-1.1%
Chronic Lung Disease (Curr)	4.3%	12.2%	-7.9%	-14.6%	-1.1%
HbA1c (Curr)	4.3%	12.2%	-7.9%	-14.6%	-1.2%
Temperature at Baseline (C) :<=/> overall median	4.4%	12.3%	-7.9%	-14.7%	-1.1%
Systolic Blood Pres at Baseline (mmHg) :<=/> overall median	4.3%	12.2%	-7.9%	-14.5%	-1.3%
Fever at Baseline Flag	4.3%	12.2%	-7.9%	-14.6%	-1.2%
Hypoxemia (Curr)	4.3%	12.3%	-8.0%	-14.7%	-1.3%
BMI Greater or Equal 28	4.2%	12.4%	-8.2%	-14.9%	-1.5%
D-Dimer (ug/ml) :<=/> overall median	3.6%	11.8%	-8.2%	-14.8%	-1.6%

Lactate Dehydrogenase (IU/L) :<=/> overall median	3.9%	12.2%	-8.3%	-15.0%	-1.6%
BMI (CRF) at Baseline (kg/m^2) :<=/> overall median	4.3%	12.6%	-8.3%	-15.2%	-1.4%
Effective SOC *	4.5%	13.1%	-8.5%	-15.7%	-1.4%
Oxygen Flow at Baseline (L/min) :<=/> overall median	4.2%	12.8%	-8.6%	-15.5%	-1.7%
C Reactive Protein (mg/L) :<=/> overall median	4.4%	13.1%	-8.7%	-15.7%	-1.7%
Lymphocytes (10^9/L) :<=/> overall median	4.0%	13.0%	-9.0%	-15.7%	-2.3%
Ferritin (ug/L) :<=/> overall median	2.9%	13.2%	-10.3%	-17.1%	-3.5%

Adjusted mortality and their difference are calculated based on the method of Direct Adjusted Survival Curves' as performed by SAS PHREG procedure. In this analysis, the confounder is entered into the model as explanatory variable and treatment group as strata variable. Each potential confounder is analysed separately. Age and number of risk factors are handled both as a continuous variable and as binary variable </>median. Overall medians are based on the overall mITT population. * Effective Standard of Care was predefined during the study and include: glucocorticoids, remdesivir and hyperimmune plasma for COVID-19.

Table S5. TESAEs by System Organ Class and Preferred Term (Safety Population).

	No. (%)	
**System Organ Class<br>Preferred Term**	**Opaganib *<br>(N=230)**	**Placebo *<br>(N=233)**
**Any TESAEs**	51 (22.2)	49 (21.0)
**Blood and lymphatic system disorders**		
**Thrombocytopenia**	0	1 (0.4)
**Cardiac disorders**	1 (0.4)	5 (2.1)
**Cardio-respiratory arrest**	1 (0.4)	2 (0.9)
**Cardiac failure acute**	0	1 (0.4)
**Cardiogenic shock**	0	1 (0.4)
**Cardiopulmonary failure**	0	1 (0.4)
**Gastrointestinal disorders**	1 (0.4)	1 (0.4)
**Gastric ulcer perforation**	1 (0.4)	0
**Abdominal wall hematoma**	0	1 (0.4)
**General disorders and administration site conditions**	2 (0.9)	1 (0.4)
**Multiple organ dysfunction syndrome**	2 (0.9)	1(0.4)
**Hepatobiliary disorders**	0	1 (0.4)
**Cholecystitis acute**	0	1 (0.4)
**Infections and infestations**	21 (9.1)	20 (8.6)
**Pneumonia**	10 (4.3)	6 (2.6)
**Septic shock**	5 (2.2)	6 (2.6)
**Sepsis**	2 (0.9)	7 (3.0)
**COVID-19 pneumonia**	1 (0.4)	2 (0.9)
**Cellulitis**	1 (0.4)	0
**Esophageal candidiasis**	1 (0.4)	0
**Pneumonia bacterial**	1 (0.4)	1 (0.4)
**Pulmonary sepsis**	1 (0.4)	1 (0.4)

**Bacterial infection**	0	1 (0.4)
**Candida infection**	0	1 (0.4)
**Investigations**	0	1 (0.4)
**Oxygen saturation decreased**	0	1 (1.4)
**Nervous system disorders**	0	4 (1.7)
**Cerebral thrombosis**	0	1 (0.4)
**Cerebrovascular accident**	0	1 (0.4)
**Depressed level of consciousness**	0	1 (0.4)
**Hemorrhagic stroke**	0	1 (0.4)
**Ischemic stroke**	0	1 (0.4)
**Psychiatric disorders**	0	1 (0.4)
**Delirium**	0	1 (0.4)
**Renal and urinary disorders**	6 (2.6)	5 (2.1)
**Acute kidney injury**	4 (1.7)	1 (0.4)
**Renal impairment**	2 (0.9)	4 (1.7)
**Respiratory, thoracic and mediastinal disorders**	36 (15.7)	39 (16.7)
**Respiratory failure**	18 (7.8)	16 (6.9)
**Acute respiratory failure**	10 (4.3)	13 (5.6)
**Pulmonary embolism**	7 (3.0)	5 (2.1)
**Acute respiratory distress syndrome**	2 (0.9)	5 (2.1)
**Pneumothorax**	2 (0.9)	0
**Bronchospasm**	1 (0.4)	0
**Hypoxia**	1 (0.4)	2 (0.9)
**Hemoptysis**	0	1 (0.4)
**Pulmonary oedema**	0	2 (0.9)
**Vascular disorders**	2 (0.9)	3 (1.3)
**Deep vein thrombosis**	1 (0.4)	1 (0.4)
**Shock**	1 (0.4)	0
**Femoral artery embolism**	0	1 (0.4)
**Hemodynamic instability**	0	1 (0.4)

* Patients are counted only once in each system organ class category, and only once in each preferred term category.

Table S6. TEAES with an Outcome of Death, SoC and Preferred Term by Treatment Group with and without Site 114.

**System Organ Class** **Preferred Term**	**Opaganib mITT * (N=230)**	**Opaganib with site 114 removed * (N=218)**	**Placebo mITT * (N=233)**	**Placebo with site 114 removed * (N=230)**
Any TEAEs	36 (15.7)	29 (13.3)	40 (17.2)	39 (16.9)
Cardiac disorders	0		4 (1.7)	
Cardiac failure acute	0		1 (0.4)	
Cardio-respiratory arrest	0		1 (0.4)	
Cardiogenic shock	0		1 (0.4)	
Cardiopulmonary failure	0		1 (0.4)	
General disorders and administration site conditions	2 (0.9)		1 (0.4)	
Multiple organ dysfunction syndrome	2 (0.9)		1 (0.4)	
Infections and infestations	16 (7.0)	9 (4.1)	12 (5.2)	11 (4.8)

Pneumonia	10 (4.3)	3 (5.2)	3 (1.3)	2 (0.8)
Septic shock	4 (1.7)		3 (1.3)	
Pulmonary sepsis	1 (0.4)		0	
Sepsis	1 (0.4)		4 (1.7)	
COVID-19 pneumonia	0		1 (0.4)	
Pneumonia bacterial	0		1 (0.4)	
	0		1 (0.4)	
Investigations				
Oxygen saturation decreased				
	0		1 (0.4)	
	0		3 (1.3)	
Nervous system disorders				
Cerebrovascular accident	0		1 (0.4)	
Haemorrhagic stroke				
Ischemic stroke	0		1 (0.4)	
	0		1 (0.4)	
Respiratory, thoracic, and mediastinal	18 (7.8)		18 (7.7)	
disorders	9 (3.9)		8 (3.4)	
Respiratory failure	3 (1.3)		2 (0.9)	
Pulmonary embolism				
Acute respiratory distress syndrome	2 (0.9)		4 (1.7)	
Acute respiratory failure	2 (0.9)		3 (1.3)	
Pneumothorax				
Pulmonary oedema	2 (0.9)		0	
	0		1 (0.4)	
Vascular disorders	0		1 (0.4)	
Femoral artery embolism	0		1 (0.4)	

*Patients are counted only once in each system organ class category, and only once in each preferred term category. Worsening of COVID-19 pneumonia was coded to COVID-19 pneumonia.
